# Exploring the therapeutic landscape of pulmonary hypertension associated with interstitial lung disease, with a focus on idiopathic pulmonary fibrosis: a narrative review

**DOI:** 10.3389/fphar.2026.1865787

**Published:** 2026-07-23

**Authors:** Nicolò Reccardini, Beatrice Da Re, Lucrezia Mondini, Francesco Salton, Marco Confalonieri, Paola Confalonieri, Michael Hughes, Giorgio Monteleone, Barbara Ruaro

**Affiliations:** 1 Department of Medical, Surgical and Health Sciences, University of Trieste, Trieste, Italy; 2 Department of Pulmonary Rehabilitation, Azienda Sanitaria Universitaria Friuli Centrale, Udine, Italy; 3 Department of Pulmonology, Santa Maria Degli Angeli Hospital, Pordenone, Italy; 4 Department of Pulmonology, Spedali Civili, Brescia, Italy; 5 Department of Pulmonology, University Hospital of Cattinara, Trieste, Italy; 6 Centre for Musculoskeletal Research, Division of Musculoskeletal and Dermatological Science, School of Biological Sciences, Faculty of Biological Medicine and Health, The University of Manchester, Manchester Academic Health Science Centre, Manchester, United Kingdom; 7 Department of Rheumatology, Salford Care Organisation, Northern Care Alliance NHS Foundation Trust, Salford, United Kingdom; 8 NIHR Manchester Biomedical Research Centre, Manchester University NHS Foundation Trust, Manchester, United Kingdom; 9 Department of Pneumology, Ruhrlandklinik, University Hospital Essen, University of Duisburg-Essen, Essen, Germany

**Keywords:** idiopathic interstitial pneumonia (IIP), idiopathic pulmonary fibrosis (IPF), PH-IPF, PH-IPF treatment, pulmonary hypertension (PH), pulmonary hypertension in interstitial lung disease (PH-ILD)

## Abstract

Pulmonary hypertension associated with idiopathic pulmonary fibrosis (PH-IPF) is a frequent and clinically relevant complication that worsens exercise capacity, quality of life, and survival. This narrative review summarizes the epidemiology, pathophysiology, diagnostic approach, and therapeutic landscape of PH-IPF. The development of PH in IPF reflects the combined effects of fibrotic parenchymal destruction, pulmonary vascular remodeling, hypoxic vasoconstriction, endothelial dysfunction, and altered vascular signaling. Diagnosis remains challenging because symptoms often overlap with those of advanced fibrotic lung disease and non-invasive tools have limited sensitivity; right heart catheterization remains the diagnostic gold standard. Antifibrotic agents are central to IPF management but have no established role as PH-targeted therapies. Most pulmonary arterial hypertension therapies have failed to show benefit in PH-IPF or have raised safety concerns, with ambrisentan and riociguat associated with harm. Inhaled treprostinil is currently the only approved therapy with randomized evidence of efficacy in PH associated with interstitial lung disease, including IPF. Supportive care, optimization of comorbidities, referral to expert centers, and timely lung transplantation evaluation remain essential components of management.

## Introduction

Idiopathic pulmonary fibrosis (IPF) is a chronic, progressive fibrosing interstitial pneumonia of unknown etiology, characterized by radiological and histological features of usual interstitial pneumonia (UIP) (Raghu et al., 2022; Ryerson et al., 2025). It is a rare disease, with an estimated global adjusted incidence and prevalence of 0.09–1.30 and 0.33 to 4.51 per 10,000 people respectively, mainly affecting older males with a history of smoking (Maher et al., 2021). The pathogenesis of IPF is primarily driven by aberrant wound healing in a genetically predisposed and aging lung, in which repeated micro-injuries to the alveolar epithelium affecting senescent type 2 alveolar epithelial cells lead to the release of profibrotic mediators and the activation of fibroblasts and myofibroblasts, which are responsible for the excessive deposition of extracellular matrix and the formation of fibroblastic foci (Lederer and Martinez, 2018; Pardo and Selman, 2021). In the correct clinical context, IPF is a multidisciplinary diagnosis based on suggestive radiological findings (definite UIP or probable UIP on high-resolution computed tomography [HRCT]) or on confirmatory histological findings in cases of inconclusive radiology (indeterminate UIP) (Raghu et al., 2022). The irreversible fibrotic remodeling of the lung parenchyma and the progressive nature of the disease lead to a gradual decline in lung function and associated quality of life, with a median survival of 3–5 years, unless the burden of disease and overall mortality are not aggravated by superimposed comorbidities such as pulmonary hypertension (PH) (Nathan and Lee, 2024; Jovano et al., 2022). In this clinical setting, the only medications approved for the primary treatment of IPF are the antifibrotics pirfenidone and nintedanib, which proved to be effective in prolonging progression-free survival, decreasing the number of acute exacerbations and, in more recent registry analyses and observational studies, reducing the risk of mortality (Nathan and Lee, 2024; Behr et al., 2020; Moon et al., 2021; Reccardini et al., 2024).

On the other hand, PH is a hemodynamic condition characterized by abnormally elevated pulmonary vascular pressures and defined by a mean pulmonary arterial pressure (mPAP) greater than 20 mmHg at rest, as assessed by right heart catheterization (Humbert et al., 2023). The estimated prevalence in the global population is 1%, with a significant increase from the age of 60 onwards. The two main causes are left heart disease and chronic obstructive pulmonary disease (COPD) respectively, but regardless of the underlying condition, the development of PH is associated with clinical deterioration and an increased risk of mortality (Humbert et al., 2023; Hoeper et al., 2016). PH is clinically classified into five groups (group 1, pulmonary arterial hypertension [PAH]; group 2, PH associated with left heart disease; group 3, PH associated with left heart disease and/or hypoxia; group 4, PH associated with pulmonary artery obstructions; group 5, PH with unclear and/or multifactorial mechanisms), which reflect different pathogenic mechanisms and influence prognosis (Humbert et al., 2023). Treatment strategies and drug availability depend largely on the etiology (and consequently on the clinical group), with vasoactive agents forming the cornerstone of treatment in PAH, while optimizing therapy for the underlying condition is the main strategy for the other classes, although prospects are constantly evolving (Humbert et al., 2023; Azaredo et al., 2024; Ruaro et al., 2022a; Ruaro et al., 2022b).

Within group 3 PH, whilst COPD represents the most common underlying condition, ILDs, including IPF, are usually associated with poorer outcomes. In this context, with increasing prevalence due to improved diagnostic techniques and the constant focus of research on offering new therapeutic perspectives, the aim of this work is to explore the landscape of therapies for PH in IPF.

### Pulmonary hypertension in idiopathic pulmonary fibrosis

#### Epidemiology and clinical impact

Reported prevalence of PH in IPF is difficult to estimate because it varies widely depending on population, diagnostic method, hemodynamic thresholds and the different stages of IPF itself. Moreover, most of the available epidemiological data on PH-IPF come from studies conducted before recent changes to the mPAP threshold and pulmonary vascular resistance (PVR) requirement (in the 2022 ESC/ERS guidelines, the mPAP threshold was lowered from ≥25 mmHg to >20 mmHg, whilst the PVR threshold for pre-capillary PH was lowered from ≥3 WU to >2 WU) (Humbert et al., 2023; Shlobin et al., 2024; Nathan et al., 2019a). Nevertheless, PH in IPF is relatively common and it can occur at any stage of the disease, but its prevalence increases in advanced stages: a mPAP ≥25 mmHg was reported in 8%–15% of patients at initial assessment, rising to 30%–50% in advanced disease and >60% in end-stage disease, reaching 86% at the time of transplantation on the United States waiting list (Shlobin et al., 2024; Nathan et al., 2019a; Lawrence et al., 2024; Raghu et al., 2015a; Kimura et al., 2013; Raghu et al., 2015b; Shorr et al., 2007; George et al., 2019).

Compared with IPF alone, PH-IPF has been associated with higher mortality, a greater risk of acute exacerbations and a more marked decline in lung function (Raghu et al., 2015a; Qiu et al., 2018; Ryerson et al., 2015). Additionally, the correlation between mPAP and survival is linear, with higher pressures associated with greater mortality (Lettieri et al., 2006). King and Nathan reported a 1-year mortality of 28% in PH-IPF versus 5.5% in those without PH, with variability correlating to severity (King and Nathan, 2017). While Raghu et al., in a cohort of 494 patients with IPF, compared individuals with group 3 PH to group 2 PH, no PH with elevated pulmonary artery wedge pressure (PAWP), and no PH with normal PAWP (Raghu et al., 2015b). Group 3 patients showed a lower percent predicted DLCO and shorter 6-minute walk distance (6MWD) than those without PH and normal PAWP, and both lower resting oxygen saturation and lower minimum oxygen saturation during the 6MWD test compared with all other groups (Raghu et al., 2015b). Additionally, among group 3 PH, PH-IPF has been associated with the worst prognosis and survival (Pescatore et al., 2024).

#### Pathophysiology

PH pathogenesis in IPF is complex, resulting from multiple and interrelated mechanisms. (i) Progressive fibrotic remodeling of the lung leads to destruction of the alveolar–capillary interface, reducing the pulmonary vascular surface and increasing PVR due to redistribution of the cardiac output in the remaining capillary bed (Cuttica, 2016; Corte et al., 2009; Pitsiou et al., 2011; Rajagopal et al., 2021). (ii) Alveolar hypoxia due to ventilation–perfusion mismatch induces hypoxic pulmonary vasoconstriction. When alveolar hypoxia is chronic, prolonged vasoconstriction ultimately leads to vascular remodeling and permanent narrowing through the release of key mediators such as hypoxia-inducible factor 1 alpha (HIF-1α), endothelin-1 (ET-1) and adenosine (Rajagopal et al., 2021; Ruffenach et al., 2020; Bryant et al., 2016; Garcia-Morales et al., 2016; Ball et al., 2014). (iii) Pulmonary vascular remodeling affects small arteries, arterioles, and veins and is characterized by intimal fibrosis and/or proliferation, medial thickening, adventitial and perivascular fibrosis, and proliferation of smooth muscle in the vascular wall. Classically, vascular remodeling is irregular both in its location within the lung (with more extensive alterations in areas where fibrosis is more advanced) and in its structural alterations. In some cases, lesions may resemble those observed in other forms of pulmonary vascular disease, although classic plexiform arteriopathy is not a defining feature of PH-IPF (Sakao et al., 2006, Rajagopal et al., 2021; Farkas et al., 2011; Pugliese et al., 2015; Parra et al., 2005). (iv) Endothelial dysfunction promotes an unbalanced release of endogenous vasoactive molecules and prothrombotic signals. The secretion of vasodilators such as nitric oxide and prostacyclin is reduced, while that of vasoconstrictors such as ET-1, thromboxane A2 and angiotensin II is increased (Farkas et al., 2011; Parra et al., 2005; Budhiraja et al., 2004). (v) The deposition of fibrotic tissue also causes architectural distortion of the vessels through compression and abnormal angulation, as well as creating anastomoses between the alveolar capillaries and the pulmonary veins (Farkas et al., 2011; Mondoni et al., 2024). (vi) The imbalance between angiogenic and angiostatic factors, growth factors and profibrotic signaling pathways also contribute to the development of PH. Endothelial-to-mesenchymal transition (EMT) is the process by which cells lose their endothelial characteristics and acquire a mesenchymal phenotype; in IPF, it has been linked to arterial remodeling and the resulting physiological changes that lead to PH (Gaikwad et al., 2022; Gaikwad et al., 2023). Vascular endothelial growth factor (VEGF) is downregulated in the fibrotic areas of IPF patients and associated with increased endothelial cells (EC) apoptosis (Farkas et al., 2009). An imbalance in the signaling of the bone morphogenetic protein receptor type 2 (BMPR2) and transforming growth factor β (TGF-β) is associated with the pathogenesis of both pulmonary fibrosis and vascular remodeling (Mondoni et al., 2024). (vii) Lastly, pulmonary microthrombosis secondary to vascular inflammation and alterations in endothelial physiology contributes to vascular obstruction and has an impact on PVR (Ryu et al., 2007).

On the contrary, PAH is a progressive distal pulmonary arterial vasculopathy driven by endothelial dysfunction, vasoconstrictor–vasodilator imbalance, smooth muscle and endothelial proliferation, and obstructive remodeling, leading to typical lesions that include medial hypertrophy, concentric intimal fibrosis, and plexiform arteriopathy (Humbert et al., 2023). Group 2 PH results from left heart disease, initially through passive transmission of elevated left-sided filling pressures, whereas in advanced stages the persistently elevated pressures may induce endothelial dysfunction and vascular remodeling (Humbert et al., 2023). PH associated to COPD and/or hypoxia primarily arises from sustained hypoxic vasoconstriction, HIF-1α mediated remodeling, endothelial dysfunction, inflammation, and capillary bed loss (Humbert et al., 2023). Chronic thromboembolic pulmonary hypertension (CTEPH) is caused by unresolved thromboembolic obstruction with fibrotic organization of the thrombi within the pulmonary arteries and its progression is magnified by secondary small-vessel arteriopathy and remodeling (Humbert et al., 2023). Group 5 PH includes multifactorial disorders (such as hemolytic anemia, myeloproliferative disease, sarcoidosis, and chronic kidney disease). In these conditions, PH reflects variable combinations of thrombosis, vascular obstruction, endothelial dysfunction, high-output states, venous involvement, and parenchymal or extrinsic vascular compromise (Humbert et al., 2023).

#### Diagnosis

The diagnosis of PH-ILD follows the standard diagnostic approach for PH, and although several non-invasive methods have been proposed for predicting PH-ILD, none of them has been standardized or widely adopted (Humbert et al., 2023; Lawrence et al., 2024). Dyspnea, which is predominantly exertional and may be disproportionate or progress at a faster rate than the parenchymal fibrotic involvement, is the main symptom reported by patients (Dumitrescu et al., 2017). Physical examination typically reveals the ‘Velcro-like’ auscultatory crackles associated with IPF, in addition to the usual signs of PH and possible signs of chronic hypoxemia (Rich et al., 1987; Condliffe et al., 2023). Pulmonary function tests usually show a restrictive impairment associated with reduced diffusing capacity of the lungs for carbon monoxide (DLCO). A disproportionate reduction in DLCO, severe exertional desaturation, reduced 6MWD, elevated BNP/Nt-proBNP, right heart enlargement, or pulmonary artery enlargement on CT scan should raise suspicion of PH in IPF, particularly when symptoms appear out of proportion to the extent of parenchymal disease. In PH-ILD, DLCO may be more severely reduced relative to forced vital capacity (FVC), although in end-stage IPF or in specific forms such as combined pulmonary fibrosis and emphysema (CPFE), this feature may be masked (Sun et al., 2003; Hoeper et al., 2022). Imaging studies, in particular chest X-rays and HRCT, reveal the classic signs of ILD alongside signs of PH (Remy-Jardin et al., 2021; Ascha et al., 2017; Hoeper et al., 2013).

Transthoracic echocardiography (TTE) remains the first-line, non-invasive screening tool, as per PH guidelines (Humbert et al., 2023). However, Keir et al. compared the modified screening criteria for PH based on TTE in accordance with the ESC/ERS guidelines with RHC in a cohort of 265 patients diagnosed with ILD and suspected PH (Keir et al., 2018). In this context, tricuspid regurgitation velocity led to misclassification as low-probability PH in 40% of patients who were subsequently diagnosed with PH by RHC, suggesting that TTE alone may not be sufficiently robust to confidently exclude PH in ILD (Keir et al., 2018; Galiè et al., 2015).

RHC is the gold standard for diagnosing and classifying PH-IPF, which is characterized by mPAP >20 mmHg, PAWP ≤15 mmHg and PVR >2 WU (Humbert et al., 2023). Within group 3 PH, patients with PVR >5 WU are further identified as a group strongly associated with a poorer prognosis and reduced survival (Humbert et al., 2023; Olsson et al., 2021).

### Antifibrotic treatment and supportive care

#### Antifibrotic drugs: pirfenidone, nintedanib, and nerandomilast

Until recently, pirfenidone and nintedanib were the only approved disease-modifying treatments for IPF. Following the FDA approval of nerandomilast, the antifibrotic landscape is expanding; however, none of these agents is specifically approved for the treatment of PH-IPF (Raghu et al., 2022). Both pirfenidone and nintedanib have been shown to be effective in slowing down fibrotic remodeling, and emerging evidence suggests that they may have beneficial effects on pulmonary vascular remodeling through mechanisms distinct from their antifibrotic properties (Tahara et al., 2019; Tsutsumi et al., 2019; Poble et al., 2019).

Pirfenidone demonstrated beneficial effects only in preclinical models. In sugen/hypoxia rat models, it effectively reduced PVR, vascular remodeling and the proliferation of pulmonary artery smooth muscle cells, whilst *in vitro* it demonstrated the ability to inhibit the NADPH/ROS/p38 pathway associated with hypoxia and to limit IL-11-induced vascular remodeling (Poble et al., 2019; Zhang et al., 2020; Roger et al., 2024).

Similarly, nintedanib reduced PVR, vascular remodeling and smooth muscle proliferation by inhibiting EMT in rat models and attenuated the vascular effects of IL-11 in *in vitro* studies, although data from clinical trials remain conflicting (Tahara et al., 2019; Tsutsumi et al., 2019; Roger et al., 2024; Kolb et al., 2018). No difference in primary endpoints of disease progression and quality of life was met even when antifibrotics were combined with sildenafil, and under current guidelines their role remains limited to management of the underlying fibrotic disease (Kolb et al., 2018; Behr et al., 2021; Olsson et al., 2023).

#### Supportive care

Supportive care for IPF includes non-pharmacological interventions, symptom management and palliative care approaches such as supplemental oxygen, pulmonary rehabilitation and optimization of comorbidities to improve quality of life (Mullholand et al., 2025).

Supplementary oxygen is not approved as a pulmonary vasodilator therapy, and the 2022 ESC/ERS guidelines restrict its use to optimizing treatment of the underlying lung condition, as the cornerstone of management for group 3 PH (Humbert et al., 2023). Although long-term oxygen therapy (LTOT) and ambulatory oxygen therapy have a theoretical role in counteracting hypoxia-induced vasoconstriction, their potential role in preventing or mitigating the development of PH remains unclear (Olsson et al., 2023; Jacobs et al., 2020). In a recent small-scale randomized trial on pre-capillary PH (not specific to IPF), Benjamin et al. reported that LTOT for 12 weeks significantly improved the 6MWD compared with controls, but no other significant results were observed regarding relevant outcomes, such as TTE and RHC measurements (Benjamin et al., 2024).

Pulmonary rehabilitation has been shown to improve exercise capacity, reduce shortness of breath and enhance quality of life; it is also essential to identify and treat comorbidities, including pulmonary embolism, obstructive sleep apnea (OSA) and gastro-esophageal reflux disease (Humbert et al., 2023; Olsson et al., 2023; Mullholand et al., 2025). None of these treatments has a direct application in PH treatment beyond optimal management of IPF (Humbert et al., 2023).

### Pulmonary arterial hypertension targeted therapies

**FIGURE 1:**
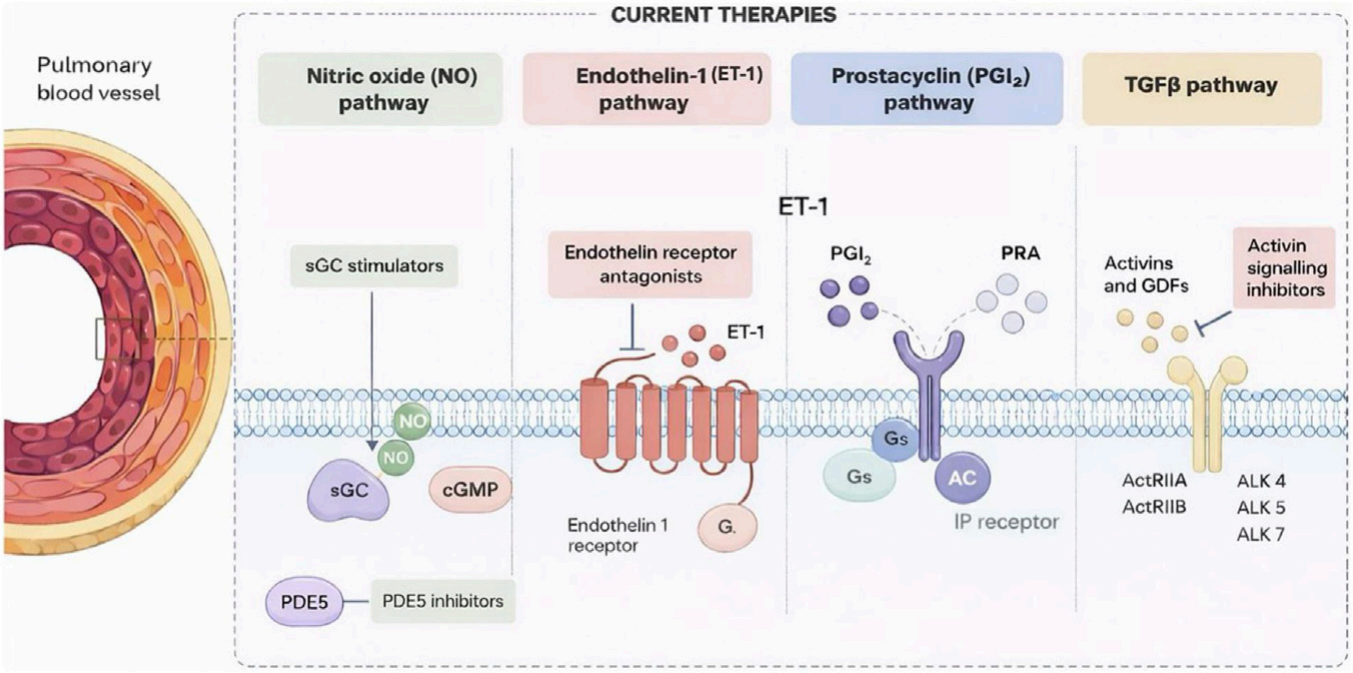
Current pulmonary arterial hypertension therapies and associated pathways. Abbreviations: AC, adenylyl cyclase; ActRIIA/IIB, activin receptor type IIA/IIB; ALK, activin receptor-like kinase; cGMP, cyclic guanosine monophosphate; ET-1, endothelin-1; G, G protein; GDFs, growth differentiation factors; Gs, stimulatory G protein; IP, I prostanoid; NO, nitric oxide; PDE5, phosphodiesterase-5; PGI2, prostaglandin I2; PRA, prostacyclin receptor agonists; sGC, soluble guanylate cyclase; TGFβ, transforming growth factor beta.

PAH treatment is based on medications targeting four main pathobiological pathways: the nitric oxide-cGMP pathway with phosphodiesterase-5 inhibitors (PDE5i) and guanylate cyclase stimulators (sGCs), the endothelin pathway with endothelin receptor antagonists (ERAs), the prostacyclin pathway with prostacyclin analogues (PCA) and prostacyclin receptor agonists (PRA), and the activin pathway with activin-signaling inhibitors (Figure 1) (Humbert et al., 2023; Ghofrani et al., 2025).

#### Phosphodiesterase-5 inhibitors and guanylate cyclase stimulators

Sildenafil and tadalafil are not recommended for routine use in PH-IPF, as numerous randomized controlled trials, including those evaluating combination therapies, have failed to demonstrate efficacy on primary endpoints (Barnes et al., 2019). However, secondary outcomes from the STEP-IPF study showed modest improvements in DLCO, oxygenation at rest, dyspnea and quality of life among patients treated with sildenafil monotherapy compared with placebo, whilst a pre-specified post-hoc analysis demonstrated that sildenafil preserved 6MWD in the subgroup of patients with right ventricular systolic dysfunction (Idiopathic Pulmonary Fib rosis Clinical Research Network et al., 2010; Han et al., 2013). The data on the use of tadalafil in group 3 PH come mainly from patients with COPD, in whom it has not shown any significant benefit (Goudie et al., 2014). Two observational studies suggested potential benefit of PDE5i in selected populations, where sildenafil and tadalafil were associated with improved hemodynamics and survival (Zimmermann et al., 2014; Dawes et al., 2023). A recent meta-registry analysis of 940 ILD-PH patients found that PDE5i treatment was associated with improved survival in patients with severe PH (defined as PVR >5 WU), with hazard ratios (HR) of 0.537 for idiopathic interstitial pneumonia (IIP)-PH, 0.461 for IPF-PH, and 0.435 for IIP-PH on antifibrotic therapy (Yogeswaran et al., 2025). Nonetheless, given the lack of robust evidence, the 2022 ESC/ERS guidelines recommend that patients with group 3 severe PH be referred to a PH center for individualized decision-making, where PDE5i may be considered (Humbert et al., 2023).

Riociguat is contraindicated in PH-IPF due to evidence of harm from the RISE-IIP trial, which demonstrated increased serious adverse events, increased mortality, and no primary outcome efficacy, leading to early termination of the study (Olsson et al., 2023; Nathan et al., 2019b). A post hoc analysis of the same study, which examined the available HRCT scans, revealed that 28% of patients had CPFE and that this condition was associated with higher mortality, suggesting that a high burden of pulmonary parenchymal disease and the presence of more extensive emphysema than fibrosis may have predisposed patients to adverse outcomes in the RISE-IIP study (Nathan et al., 2021a). The 2022 ESC/ERS guidelines explicitly do not recommend the use of riociguat in PH-IIP (Humbert et al., 2023).

#### Endothelin receptor antagonists

ERAs are contraindicated in PH-IPF, based on multiple clinical trials demonstrating lack of efficacy and, in the case of ambrisentan, actual evidence of harm (Olsson et al., 2023). The ARTEMIS-IPF trial evaluated ambrisentan versus placebo in IPF but was terminated early due to evidence of increased disease progression, respiratory hospitalizations and deaths in the treatment arm, with similar results in the PH subgroup (Raghu et al., 2013a). Based on these results and the similar trend that was emerging, the ARTEMIS-PH trial (NCT00879229) was also terminated early. Although bosentan was well tolerated in patients with IPF, it failed to improve the hemodynamic parameters measured invasively in patients with IIP and PH compared with placebo (Corte et al., 2014; King et al., 2011). The use of macitentan in PH-ILD has never been investigated in large randomized trials. In the MUSIC trial, it failed to meet either the primary or secondary endpoints and demonstrated a safety profile similar to that of placebo in patients with IPF, although the trial did not systematically assess PH or report any subgroup data on patients with PH (Raghu et al., 2013b). A preclinical study conducted on rat models with pulmonary fibrosis suggested that macitentan might reduce both mPAP and the progression of fibrosis, but to date these results have not been replicated in human clinical trials (Bellaye et al., 2018). Current guidelines do not recommend for routine use of ERAs in PH-IPF (Humbert et al., 2023).

### Prostacyclin analogues and prostacyclin receptor agonists

Inhaled treprostinil is the only PCA and PAH-specific therapy approved for PH-ILD, including PH-IPF, based on the positive results of the INCREASE trial (Humbert et al., 2023; Waxman et al., 2021). In a population with ILD consisting mainly of patients with IIP, inhaled treprostinil, compared with placebo, demonstrated a significant improvement in 6MWD, a reduction in N-terminal brain natriuretic peptide (NT-proBNP) and a lower incidence of clinical worsening (primarily hospitalizations for cardiopulmonary indication), with the results of the open-label extension study confirming long-term safety and efficacy (Waxman et al., 2021; Waxman et al., 2023). A post hoc analysis of both the INCREASE trial and its extension study reported a non-significant benefit in terms of long-term survival (HR 0.71; 95% confidence interval [CI] 0.46 to 1.10; p = 0.1227) using the conventional analysis; however, when two different survival models were applied, inhaled treprostinil demonstrated a significant reduction in mortality (HRs of 0.62; 95% CI 0.39 to 0.99; p = 0.0483 and 0.26; 95% CI 0.07 to 0.98; p = 0.0473) (Nathan et al., 2024). Furthermore, post hoc analyses showed that higher doses of inhaled treprostinil were associated with greater benefits in terms of both preventing clinical deterioration and promoting clinical improvement; that patients in the treatment group had a greater win ratio related to death from any cause, cardiopulmonary hospitalizations, 6MWD decline and clinical improvement compared to placebo; that inhaled treprostinil was associated with a significantly lower risk of further disease progression following an initial episode of clinical worsening (Nathan et al., 2023a; Nathan et al., 2023b; Nathan et al., 2022). In another post-hoc analysis of the INCREASE trial, it was associated with an improvement in FVC, with particularly marked results in patients with IPF, and the recent TETON-2 study, which evaluated its use in patients with IPF regardless of their PH status, demonstrated a significantly smaller decline in FVC and fewer episodes of clinical worsening compared with placebo (Nathan et al., 2021b; Nathan et al., 2026). These findings support the hypothesis that inhaled treprostinil may have disease-modifying effects in IPF, although the mechanisms and their relationship with antifibrotic activity require further clarification. Established possible adverse effects of inhaled treprostinil are symptomatic hypotension (due to systemic vasodilation) and increased bleeding (via inhibition of platelet aggregation), nevertheless, in the INCREASE trial, the most common adverse events were cough, headache, dyspnea, dizziness, nausea, and fatigue, with a safety profile similar to prior studies (Waxman et al., 2021).

Inhaled iloprost shares the same mechanisms of action as inhaled treprostinil, although its pharmacokinetics differ slightly, particularly regarding half-lives (20–30 min and 4.5 h, respectively) (Burger et al., 2023). However, it has not been studied in randomized controlled trials for PH-IPF or PH-ILD, and most of the evidence supporting its use in group 3 PH associated with interstitial lung disease comes from small studies and case reports. In 1999, Olschewski et al. reported a significant reduction in mPAP and PVR associated with aerosol iloprost treatment compared with other vasodilator therapies, in a cohort of 8 patients with pulmonary fibrosis and severe PH (Olschewski et al., 1999). A subsequent randomized placebo-controlled trial evaluating the safety of inhaled iloprost in PH-IPF concluded that, although no safety risks were apparent, no clinical benefit in terms of efficacy was observed (Krowka et al., 2007). Recently, in a single case report, inhaled iloprost was associated with improved mPAP, PVR, 6MWD, FVC and DLCO in a patient with severe PH-IPF (Jang et al., 2024).

At present, inhaled iloprost is not approved for PH-IPF, and its use should be regarded as unsupported by robust evidence outside individualized expert-center decision-making or research settings (Humbert et al., 2023; Shlobin et al., 2024).

Inhaled PCA offer theoretical advantages over systemic administration, as they are preferentially delivered to the ventilated lung units, where blood flow is redirected, thereby reducing the ventilation-perfusion mismatch. In contrast, systemic PCA are associated with concerns regarding worsening ventilation-perfusion mismatch, as are other oral therapies for PAH. For this reason and based on the documented potential risks associated with ambrisentan and riociguat, systemic PCA (such as epoprostenol) and PRA (such as selexipag) have not been specifically studied in PH-ILD and are not recommended for routine use in this population (Humbert et al., 2023; Shlobin et al., 2024; Ghofrani et al., 2002).

#### Activin-signaling inhibitors

Sotatercept is the first agent in this category approved for PAH and, to date, has not been studied in PH-ILD or PH-IPF (Hoeper et al., 2023). In a small observational study involving 7 patients with PAH associated with connective tissue disease and concomitant ILD, the addition of sotatercept to standard PAH therapy was associated with an improvement in hemodynamic, clinical and laboratory parameters, although the study lacked a control group (Dagher et al., 2025). These data should not be extrapolated to PH-ILD or PH-IPF, because the study population was primarily PAH associated with connective tissue disease with concomitant ILD, rather than group 3 PH.

## Lung transplantation and future perspectives

### Lung transplantation

Lung transplantation is the only definitive therapeutic option for selected patients with advanced IPF and associated PH and associated PH. According to the International Society for Heart and Lung Transplantation (ISHLT), the development of PH is a listing criterion for patients with ILD (George et al., 2019). In IPF, PH is associated with an increased risk of primary graft dysfunction and is an independent risk factor for increased 90-day mortality; however, a registry analysis of 2,542 patients with IPF found no significant differences in long-term survival based on pulmonary hypertension status (univariable HR 1.011; 95% CI 0.886 to 1.153; p = 0.8760 and multivariable HR 0.924; 95% CI 0.792 to 1.078; p = 0.3176), with a median survival of approximately 60 months for both groups (Whelan et al., 2005; Hayes et al., 2015). Nevertheless, lung transplantation itself is associated with inferior outcomes in PH-IPF than for most other indications (George et al., 2019). Based on limited medical options, poor prognosis and unpredictable progression, 2022 ESC/ERS guidelines recommend that eligible patients with lung disease and PH should be referred for lung transplantation evaluation (Humbert et al., 2023).

### Future perspectives

In October 2025 the Food and Drug Administration (FDA) approved nerandomilast, an orally administered preferential inhibitor of phosphodiesterase 4B with antifibrotic and immunomodulatory effects (Richeldi et al., 2025). By slowing down the underlying fibrotic process and reducing the expression of inflammatory cytokines that are overexpressed in IPF, nerandomilast may indirectly prevent or limit the development of PH-IPF. Frespaciguat (MK-5475, NCT05612035), an inhaled sGCs, is under active clinical investigation in adults with PH-COPD, with future potential applications in PH-ILD. Due to its inhaled administration, frespaciguat may be preferentially delivered to the ventilated areas of the lungs, thereby reducing the ventilation-perfusion mismatch. Sotatercept, which showed promising results in PAH by targeting pulmonary vascular remodeling rather than vasodilation and resulting in reverse remodeling and restoration of vessel patency, may have applications in other forms of PH, although no clinical study with this aim has yet been registered on ClinicalTrials.gov. To date, three further molecules (NCT06475781, NCT07333183, NCT07175038), at various study phases, are listed on ClinicalTrials.gov and actively recruiting, with the aim of assessing their efficacy and safety specifically in PH-ILD.

From a non-pharmacological viewpoint, emerging preclinical evidence suggests a complex role for vagal innervation in pulmonary fibrosis. Unilateral vagotomy attenuated bleomycin-induced lung fibrosis in mice by reducing collagen deposition, myofibroblast accumulation, and TGF-β signaling, suggesting that cholinergic pathways may paradoxically promote fibrotic remodeling despite their established anti-inflammatory properties in acute lung injury (Song et al., 2015). These findings raise the hypothesis that autonomic imbalance—specifically, parasympathetic overactivation—may contribute to the fibrotic milieu in PH-IPF. In this context, bioelectric medicine approaches such as targeted somatosensory nerve stimulation, which has been shown to modulate parasympathetic tone in healthy volunteers, represent a novel investigational avenue for autonomic rebalancing (Prye et al., 2025). However, no preclinical or clinical data currently link neuromodulation strategies to outcomes in pulmonary fibrosis or PH-ILD, and further translational research is needed before these concepts can be considered for therapeutic application.

### Management of PH-ILD and PH-IPF in referral centers

Finally, expert consensus statements and international guidelines consistently emphasize that patients with PH-ILD should be referred to expert centers able to confirm the diagnosis by right heart catheterization and experienced in conducting clinical trials with investigational therapies (Humbert et al., 2023; Hoeper et al., 2016; Shlobin et al., 2024; Raghu et al., 2015a).

## Discussion

This narrative review highlights the complex and multifactorial nature of PH-IPF, emphasizing both the clinical burden of this condition and the persistent therapeutic gaps that characterize its management, and reinforcing the view that PH-IPF is not merely a comorbidity, but rather a key determinant of prognosis, contributing substantially to functional decline, an increased risk of exacerbations and reduced survival.

From a pathophysiological perspective, the coexistence of fibrotic parenchymal destruction and pulmonary vascular remodeling emerges as a central theme. The interplay between loss of pulmonary vascular surface, hypoxic vasoconstriction, endothelial dysfunction, and molecular signaling pathways suggests that PH-IPF represents a distinct phenotype rather than a simple overlap between two diseases. This conceptual shift has important implications, as it supports the need for targeted therapeutic strategies that address both fibrotic and vascular components simultaneously. However, despite increasing mechanistic insights, translation into effective clinical interventions remains limited.

Non-invasive diagnostic tools, including TTE, which remain the mainstay of PH screening, may not be sufficiently sensitive in cases of PH-IPF and may underestimate the prevalence of the disease, thereby delaying the initiation of appropriate treatment. Additionally, the similar and already pathologic clinical presentation can further complicate the initial diagnostic process. The fact that RHC continues to be regarded as the gold standard highlights the need for more accurate and accessible screening strategies, particularly among at-risk and vulnerable populations, where advanced stages of the disease may limit the feasibility of invasive procedures.

In recent years, targeted therapies for PAH have substantially improved outcomes in Group 1 PH, but their effects in PH-IPF have largely been disappointing or even harmful, mainly due to the different pathogenic mechanisms and the protective role of hypoxia-induced vasoconstriction in maintaining gas exchange (Table 1). ERAs and sGCs, for instance, have consistently failed to demonstrate benefit and, in some cases, have been associated with increased morbidity and mortality. This highlights the potential risk of extrapolating treatment paradigms across different PH groups without accounting for underlying pathobiological differences, particularly the presence of significant parenchymal lung disease and ventilation–perfusion mismatch.

**TABLE 1:** Current pulmonary arterial hypertension therapies and associated pathways.

Drug/Class	Mechanisms	Evidence in PH-IPF/PH-ILD	Current position	References
Pirfenidone	Inhibition of TGF-β induced pathways	Experimental models (*in vivo*) ↓ Pulmonary vascular resistance (PVR) ↓ Vascular remodeling ↓ Proliferation of pulmonary artery smooth muscle cells — (*In vitro*) ↓ Vascular remodeling	Not recommended, limited to the primary IPF management	Poble et al. (2019) Zhang et al. (2020) Roger et al. (2024) Kolb et al. (2018) Behr et al. (2021) Olsson et al. (2023)
Nintedanib	Inhibition of receptor tyrosine kinases (PDGFR, FGFR, VEGFR) and non-receptor tyrosine kinases of the Src family	Experimental models (rat/*in vitro*) ↓ PVR ↓ Vascular remodeling ↓ Smooth muscle cell proliferation Clinical studies No significant differences in primary endpoints (disease progression, quality of life)	Not recommended, limited to the primary IPF management	Tsutsumi et al. (2019) Poble et al. (2019) Roger et al. (2024) Kolb et al. (2018) Behr et al. (2021) Olsson et al. (2023)
Sildenafil/Tadalafil	Selective inhibitor of c-GMP specific-phosphodiesterase	IPF + RVSD ↓ decline in 6MWT; ↑ SGRQ STEP-IPF TRIAL (Sildenafil vs. placebo) ↑ DLCO, O2, QoL (modest) Severe PH-ILD (PVR >5 WU) ↑ Hemodynamics Group 3 PH Consider PDE5i in expert centers	Severe Group 3 PH: refer to PH center; consider PDE5 inhibitors (ESC/ERS 2022)	Humbert et al. (2023) Idiopathic Pulmonary Fib rosis Clinical Research Network et al. (2010) Han et al. (2013) Goudie et al. (2014) Zimmermann et al. (2014)
Riociguat	Stimulator of soluble guanylate cyclase	RISE IIP TRIAL ↑ Serious adverse events and mortality; no efficacy → early termination	Contraindicated in PH-IPF; not recommended in PH-IIP (ESC/ERS 2022 guidelines)	Humbert et al. (2023) Olsson et al. (2023) Nathan et al. (2019b)
Ambrisentan	Antagonist of Endothelin receptor	ARTEMIS-IPF TRIAL Early termination vs. placebo due to ↑ progression, hospitalizations, and mortality; similar PH subgroup findings	Not recommended in PH-IPF (2022 ESC/ERS guidelines)	Humbert et al. (2023) Raghu et al. (2013a)
Bosentan	Antagonist of Endothelin receptor A and B	BPHIT TRIAL No difference vs. placebo (hemodynamics, functional capacity, symptoms, 16 weeks BUILD-3 (Trial) No clinical benefit; no effect on HRQoL or dyspnea; slight ↑ FVC/DLCO; safety comparable to placebo	Not recommended in PH-IPF (2022 ESC/ERS guidelines)	Humbert et al. (2023) Corte et al. (2014) King et al. (2011)
Macitentan	Antagonist of Endothelin receptor	Preclinical studies ↓ mPAP, ↓ fibrosis in rat) not confirmed in human trials MUSIC TRIAL Failed primary/secondary endpoints; safety similar to placebo; PH not evaluated (no PH subgroup)	Not recommended in PH-IPF (2022 ESC/ERS guidelines)	Raghu et al. (2013b) Bellaye et al. (2018) Humbert et al. (2023)
Inhaled Treprostinil	Analogue of prostacyclin	INCREASE OLE TRIAL Long-term ↑/stable 6MWD; ↓ NT-proBNP; ↑ FVC; ↓ exacerbations; confirmed long-term safety/efficacy INCREASE TRIAL ↑ 6MWD; ↓ NT-proBNP; ↓ clinical worsening (↓ cardiopulmonary hospitalizations); long-term safety/efficacy confirmed Post-hoc analysis of the INCREASE TRIAL Trend toward ↓ mortality; modelling analyses suggest possible long-term survival benefit of inhaled treprostinil in PH-ILD. Post Hoc analysis of INCREASE STUDY ↑ dose-dependent benefit: ↓ clinical worsening; ↑ clinical improvement (≥9 breaths per session of inhaled treprostinil) Win Ratio of INCREASE TRIAL ↑ win ratio vs. placebo; consistent benefit on mortality, hospitalizations, 6MWD, and clinical improvement (sensitivity analyses confirm INCREASE post-hoc ↓ disease progression events; ↓ multiple events vs. placebo; benefits maintained after initial progression Post Hoc analysis INCREASE TRIAL Trend ↑ FVC (% predicted significant); IPF subgroup ↑ FVC at week 16; common AEs: cough, headache, dyspnea, GI symptoms TETON-2 TRIAL ↑ FVC vs. placebo; ↓ clinical worsening; no effect on IPF exacerbation; ↑ cough and discontinuations Retrospective study ↑ adherence and persistence vs. iloprost; ↓ hospitalizations/ED visits; similar costs	PAH-specific therapy approved for PH-ILD	Waxman et al. (2021) Waxman et al. (2023) Nathan et al. (2024) Nathan et al. (20233a) Nathan et al. (2023b) Nathan et al. (2022) Nathan et al. (2021b) Nathan et al. (2026) Burger et al. (2023)
Inhaled Iloprost	Analogue of prostacyclin	CLINICAL TRIAL Safe; no efficacy vs. placebo (6MWD, symptoms, oxygenation); no proven benefit in IPF-PH. CLINICAL TRIAL ↑ selective pulmonary vasodilation with preserved gas exchange; possible life-saving benefit in decompensated PH secondary to lung fibrosis (inhaled PGI2/iloprost	• Not approved PH-IPF (consider administration for individualized treatment in PH centers)	Humbert et al. (2023) Olschewski et al. (1999) Krowka et al. (2007) Jang et al. (2024)
Epoprostenol	Analogue of prostacyclin	CLINICAL TRIAL ↓ PVR with all agents; sildenafil (and NO) preserved V/Q and ↑ oxygenation, while epoprostenol worsened V/Q mismatch; sildenafil showed preferential pulmonary vasodilation	Not recommended in PH-ILD	Ghofrani et al. (2002)
Selexipag	Selective prostacyclin receptor agonist	Not tested in PH-ILD	Not recommended in PH-ILD	​
Sotatercept	Activin-signaling inhibitors	Not tested in PH-ILD STELLAR TRIAL (PAH) ↑ 6MWD vs. placebo; multiple secondary endpoints improved (PVR, NT-proBNP, WHO-FC, clinical worsening); AEs: epistaxis, ↑Hb, thrombocytopenia, ↑BPApproved in PAH Retrospective Study ↑ 6MWD; ↓ PVR, eRVSP, NT-proBNP; improved WHO-FC and O2 need; well tolerated (small CTD-PAH + ILD cohort)	Not recommended in PH-ILD	Hoeper et al. (2023) Dagher et al. (2025)
Nerandomilast	Inhibitor of PDE4B	Not tested in PH-ILD	Not recommended, limited to the IPF and PPF treatment	Richeldi et al. (2025) Maher et al. (2025)

Abbreviations: PH-ILD, pulmonary hypertension associated interstitial lung disease; RVSD, right ventricular systolic disfunction; HRQOL, health-related quality of life; 6MWT, 6-min walking test; SGRQ, St. George’s Respiratory Questionnaire; FVC, forced vital capacity; mPAP, mean pulmonary artery pressure; IPF, idiopathic pulmonary fibrosis; DLCO, diffusing lung capacity for carbon monoxide; NO, nitric oxide.

In this context, inhaled treprostinil represents a major therapeutic advance, as it is the only approved therapy with randomized evidence of efficacy in PH-ILD, including IPF (Humbert et al., 2023). Its inhaled route may preferentially target ventilated lung units, thereby limiting the risk of worsening ventilation–perfusion mismatch. However, its impact on long-term survival remains incompletely defined, and further real-world data are needed.

Another key aspect is the limited role of antifibrotic agents in directly addressing PH-ILD. Although pirfenidone and nintedanib remain the cornerstone of IPF management and their documented efficacy in slowing down fibrotic progression should theoretically limit PH development, their effects on pulmonary vascular remodeling are largely confined to preclinical observations or indirect clinical benefits. This reinforces the need for integrated therapeutic approaches that go beyond fibrosis control, particularly in patients with advanced disease or severe hemodynamic impairment.

Looking ahead, emerging therapies targeting novel pathways—such as activin signaling inhibitors and phosphodiesterase 4 inhibitors—offer promising avenues for future research. However, robust clinical trials designed for PH-ILD, and specifically PH-IPF, populations are essential to validate their efficacy and safety.

Overall, PH-IPF remains a high-risk phenotype characterized by substantial, unmet diagnostic and therapeutic needs. The available evidence does not support the indiscriminate use of PAH-targeted therapies in this setting, and some agents may be harmful. Inhaled treprostinil has changed the therapeutic landscape by providing randomized evidence of benefit in PH-ILD, but several questions remain, including optimal patient selection, timing of initiation, long-term safety, survival impact, and integration with antifibrotic or emerging disease-modifying therapies. Given the diagnostic complexity, the need for right heart catheterization, and the potential risks of inappropriate vasodilator therapy, patients with suspected or confirmed PH-IPF should be referred to expert centers with experience in ILD, pulmonary vascular disease, invasive hemodynamic assessment, clinical trials, and lung transplantation pathways.

## Glossary

BMPR2: bone morphogenic protein receptor type 2
CI: confidence interval
COPD: chronic obstructive pulmonary disease
CPFE: combined pulmonary fibrosis and emphysema
CTEPH: chronic thromboembolic pulmonary hypertension
EC: endothelial cells
EndMT: endothelial-to-mesenchymal transition
ERAs: endothelin receptor antagonists
ET-1: endothelin 1
FDA: Food and Drug Administration
FVC: forced vital capacity
HR: hazard ratio
HIF-1α: hypoxia-inducible factor 1 alpha
HRCT: high resolution computed tomography
IIP: idiopathic interstitial pneumonia
IPF: idiopathic pulmonary fibrosis
ISHLT: International Society for Heart and Lung Transplantation
LTOT: long-term oxygen therapy
mPAP: mean pulmonary arterial pressure
NT-proBNP: N-terminal pro-brain natriuretic peptide
PAH: pulmonary arterial hypertension
PAWP: pulmonary artery wedge pressure
PCA: prostacyclin analogues
PDE5i: phosphodiesterase-5 inhibitors
PH: pulmonary hypertension
PRA: prostacyclin receptor agonists
PVR: pulmonary vascular resistance
sGCs: soluble guanylate cyclase stimulators
TGF-β: transforming growth factor β
UIP: usual interstitial pneumonia
6MWD: 6-min walk distance
